# Vitamin D Status and Related Factors in Newborns in Shanghai, China

**DOI:** 10.3390/nu6125600

**Published:** 2014-12-04

**Authors:** Xiaodan Yu, Weiye Wang, Zhenzhen Wei, Fengxiu Ouyang, Lisu Huang, Xia Wang, Yanjun Zhao, Huijuan Zhang, Jun Zhang

**Affiliations:** 1 MOE-Shanghai Key Lab of Children’s Environmental Health, Xinhua Hospital affiliated to Shanghai Jiao Tong University School of Medicine, Shanghai 200092, China; E-Mails: wcwangw13@yahoo.com (W.W.); zq744143217@126.com (Z.W.); ouyangfx@yahoo.com (F.O.); huanglisu@gmail.com (L.H.); wxsjtu@gmail.com (X.W.); 13524813362@163.com (Y.Z.); zhangjunjim@gmail.com (J.Z.); 2 Departments of Pathology and Bio-Bank, the International Peace Maternity and Child Health Hospital, Shanghai Jiao Tong University, Shanghai 200030, China; E-Mail: zhanghj815@126.com

**Keywords:** vitamin D, related factors, newborn

## Abstract

With the increasing recognition of the importance of the non-skeletal effects of vitamin D (VitD), more and more attention has been drawn to VitD status in early life. However, the VitD status of newborns and factors that influence VitD levels in Shanghai, China, remain unclear. A total of 1030 pregnant women were selected from two hospitals in Shanghai, one of the largest cities in China located at 31 degrees north latitude. Umbilical cord serum concentrations of 25-hydroxy vitamin D [25(OH)D] were measured by LC-MS-MS, and questionnaires were used to collect information. The median cord serum 25(OH)D concentration was 22.4 ng/mL; the concentration lower than 20 ng/mL accounted for 36.3% of the participants, and the concentration lower than 30 ng/mL for 84.1%. A multivariable logistic regression model showed that the determinants of low 25(OH)D status were being born during autumn or winter months and a lack of VitD-related multivitamin supplementation. The relative risk was 1.7 for both autumn (95% CI, 1.1–2.6) and winter (95% CI, 1.1–2.5) births (*p* < 0.05). VitD-related multivitamin supplementation more than once a day during pregnancy reduced the risk of VitD deficiency [adjusted OR (aOR) = 0.6, 95% CI (0.45–1.0) for VitD supplementation] (*p* < 0.05). VitD deficiency and insufficiency are common in newborns in Shanghai, China, and are independently associated with season and VitD supplementation. Our findings may assist future efforts to correct low levels of 25(OH)D in Shanghai mothers and their newborn children.

## 1. Introduction

VitD deficiency during pregnancy and early childhood leads to a variety of health problems for both the mother and the child [1,2,3]. Although VitD status during pregnancy has important implications regarding maternal complications, including decreased weight gain [4], gestational diabetes [5], preeclampsia [6], infections [7], and caesarean section [8], it may actually have more important implications for the general health of the developing fetus and newborn child. VitD deficiency in the newborn has been linked to hypocalcaemia, low birth weight, allergies, type I diabetes, impaired development, heart failure and rickets [9,10,11].

Over the past decade, numerous studies have reported on VitD status in adults, the elderly and, increasingly, pregnant women. However, studies regarding the prevalence of VitD deficiency among newborns are limited. In China, although there have been some reports of VitD status in pregnant women, there have only been two reports of newborns’ VitD status with a small sample size: one in Beijing (40 degrees north latitude) and one in Chengdu (30 degrees north latitude) [12,13]. Shanghai, one of the largest cities in China with more than 20 million people, is located in East China at 31 degrees north latitude. According to the only study on VitD levels in pregnant women in Shanghai, over 90.5% of these women had 25(OH)D levels below 30 ng/mL [14]. VitD supplementation of 10 μg/day during pregnancy, suggested by the Chinese Nutrition Medicine Association, was equal to the recommended amount for the adult. In addition, it was reported, although the data is limited, that the vast majority of Chinese women do not in reality take VitD supplementation during pregnancy [12]. To date, the VitD status of Shanghai newborns has not been reported.

To address this gap in the literature, we measured serum levels of 25(OH)D in the cord blood of 1030 healthy newborns in Shanghai to determine their VitD status. Having documented a high prevalence of VitD deficiency (defined as 20 ng/mL), we then examined factors that independently predicted VitD status at birth.

## 2. Experimental Methods

### 2.1. Study Design and Subjects

The Shanghai Allergy Cohort Study was a prospective study with a birth cohort of 1071 infants recruited between 2012 and 2013 at Xinhua Hospital and the International Peace Maternity and Child Hospital, two large tertiary hospitals in Shanghai. Prior to delivery, written informed consent was obtained from the mothers, and trained nurses conducted face-to-face interviews. At birth, study nurses collected the newborn’s anthropometric details and umbilical cord blood, when available. Ethics approval was obtained by the Ethics Committees of both Xinhua Hospital affiliated to Shanghai Jiao Tong University School of Medicine and the International Maternal and Children Care Hospital.

### 2.2. Umbilical Cord Blood 25(OH)D

The primary outcome measurement of the present study was the cord serum level of 25(OH)D. Cord blood was available for 1071 of the newborn participants, and 1030 of those newborns’ mothers completed the questionnaires. The cord blood samples were centrifuged and transferred to −80 °C freezers within 2 h. We used the sensitive liquid chromatography tandem mass spectrometry (LC-MS/MS) analytical method to detect serum 25(OH)D following the procedure reported by our previous study [15]. In this assay, the level of sensitivity for LC/MS/MS assay was 0.05 ng/mL for 25(OH)D_2_, and 0.1 ng/mL for 25(OH)D_3_. The serum samples (100 μL) were deproteinised and precipitated using methanol, acetonitrile, zinc sulfate, and internal standards that included deuterated 25(OH)D_2_ and 25(OH)D_3_ (Sigma, St. Louis, MO, USA). Chromatographic separations were obtained using an Agilent Poroshell 120 EC-C18 (50 × 2.1 mm, 2.7 μm) column with a gradient of water (containing 0.1% formic acid) and methanol as the mobile phase at a flow rate of 0.5 mL/min. Multiple reaction monitoring (MRM) of the analyses was performed under electrospray ionization (ESI) in the positive mode at *m/z* 401.3→383.2 and 401.3→159.1 for 25(OH)D_3_, *m/z* 413.3→395.3 and 413.3→355.2 for 25(OH)D_2_, and *m/z* 404.3→386.3 and 416.4.3→398.3 for d3-25(OH)D_3_ and d3-25(OH)D_2_, respectively. Although there is no consensus on optimal levels of 25(OH)D as measured in serum, Vit D deficiency has been historically defined and recently recommended by the Institute of Medicine (IOM) as a 25(OH)D of less than 20 ng/mL [16,17]. VitD deficiency was defined as a serum 25(OH)D concentration <20 ng/mL, and VitD insufficiency was defined as a serum 25(OH)D concentration <30 ng/mL [18,19].

### 2.3. Risk Factors

The questionnaire documented socioeconomic status, maternal age, weight and height of the mother prepregnancy, VitD and other multivitamin supplementations, and outdoor activity during pregnancy. Gestational age, newborn sex, month of birth, and birth weight were obtained from the participants’ medical records.

### 2.4. Data Analysis

Serum 25(OH)D concentrations were expressed in ng/mL. We first determined the percentiles of 25(OH)D and the prevalence of VitD deficiency in the newborns (Table 1). Then, we performed a univariate analysis to examine the correlations of 25(OH)D level with different groups of related factors (Table 2) and used multivariable analysis to estimate the independent relationship between 25(OH)D deficiency and the analyzed related factors after adjusting for potential confounders (Table 3). *P* Values of <0.05 were considered significant. All analyses were performed using Empower(R) (www.empowerstats.com, X&Ysolutions, Inc., Boston, MA, USA) and R (http://www.R-project.org).

## 3. Results

Table 1 showed the quartile values of 25(OH)D (Q1 = 18.5 ng/mL, Q2 = 22.4 ng/mL, Q3 = 27.5 ng/mL), 25(OH)D_2_ (Q1 = 3.7 ng/mL, Q2 = 4.6 ng/mL, Q3 = 5.3 ng/mL) and 25(OH)D_3_ (Q1 = 14.1 ng/mL, Q2 = 17.9 ng/mL, Q3 = 23.0 ng/mL). Participants had a median cord blood 25(OH)D concentration of 22.4 ng/mL. The 25(OH)D_3_ (Q2 = 17.9 ng/mL) concentration was higher than the 25(OH)D_2_ (Q2 = 4.6 ng/mL) concentration, and the ratio of 25(OH)D_3_:25(OH)D_2_ was 4:1. Overall, 36.3% of Shanghai newborns had serum 25(OH)D levels <20 ng/mL and 84.1% had levels <30 ng/mL (Table 1).

**Table 1:** Vitamin D level and the prevalence of vitamin D deficiency in newborns (*n* = 1030).

	25(OH)D_2_	25(OH)D_3_	25(OH)D
Q1	3.7	14.1	18.5
Q2	4.6	17.9	22.4
Q3	5.3	23.0	27.5
Min (ng/mL)	0.1	8.3	11.5
Max (ng/mL)	11.5	45.1	51.1
Mean ± SD (ng/mL)	4.5 ± 1.2	19.0 ± 6.1	23.5 ± 6.2
The prevalence of VitD deficiency (%) [25(OH)D < 20 ng/mL]	-	-	36.3
The prevalence of VitD insufficiency (%) [25(OH)D < 30 ng/mL]	-	-	84.1

The unadjusted associations between the various characteristics and VitD status were strongest for month (season) of birth and VitD related multivitamin supplementation (all *p* for trend <0.001). As expected, the median serum 25(OH)D concentrations peaked in infants born during summer months and were lower for infants born in the autumn and winter. The unadjusted analyses also indicated that outdoor activity on weekdays was a potential determinant of newborn vitamin D status (*p* < 0.05) (Table 2).

**Table 2:** The correlations of various factors with cord blood 25(OH)D by univariate analysis (*n* = 1030).

Variable	(%)	25(OH)D (Mean ± SD) (ng/mL)	*p* value
**Vitamin D category (ng/mL)**			
<20	36.3	17.5 ± 1.8	0.0000 **
≥20	63.7	26.9 ± 5.1	
**Maternal age (years)**			
<30	51.0	23.3 ± 6.2	0.2914
30–34	39.5	23.5 ± 6.2	
35–39	8.5	24.4 ± 6.0	
40+	1.0	25.5 ± 6.7	
**Maternal prepregnancy BMI**			
<28	95.3	23.5 ± 6.2	0.2292
≥28	4.7	22.4 ± 5.4	
**Maternal education**			
Middle school or lower	2.8	22.7 ± 6.2	0.7719
High school	11.5	23.5 ± 7.0	
College or higher	85.7	23.5 ± 6.1	
**Gestational age (weeks)**			
<37	3.5	23.7 ± 6.2	0.1122
37–39	71.7	23.5 ± 6.2	
40+	24.8	22.8 ± 6.1	
**Birth weight (g)**			
<2500	2.4	24.5 ± 6.9	0.4224
≥2500	97.1	23.5 ± 6.2	
Gender			
boy	50.4	23.2 ± 6.2	0.4504
girl	49.6	23.7 ± 6.3	
**Month of birth**			
Summer (Jun.–Aug.)	16.7	23.3 ± 6.1	0.0009 **
Autumn (Sep.–Nov.)	46.5	22.6 ± 6.0	
Winter (Dec.–Feb.)	36.8	22.4 ± 6.3	
**VitD supplementation during pregnancy**			
No	78.7	23.0 ± 6.1	0.0000 **
≤6 times/week	4.1	24.7 ± 6.3	
≥1 time/day	17.2	25.3 ± 6.3	
**Calcium supplementation during pregnancy**			
No	18.2	22.3 ± 5.9	0.0013 **
≤6 times/week	11.7	22.6 ± 6.0	
≥1 time/day	70.1	23.9 ± 6.3	
**DHA supplementation during pregnancy**			
No	63.1	23.0 ± 6.0	0.0024 **
≤6 times/week	6.6	24.4 ± 6.5	
≥1 time/day	30.3	24.4 ± 6.4	
**Outdoor activity in weekdays**			
<0.5 h	43.6	22.9 ± 5.9	0.0267 *
≥0.5 h	56.4	23.8 ± 6.3	
**Outdoor activity in weekend**			
<0.5 h	48.6	23.1 ± 6.1	0.1328
≥0.5 h	51.4	23.7 ± 6.3	
**Husband smoke during pregnancy**			
No	99.7	23.7 ± 6.2	0.0779
Yes	0.3	22.9 ± 6.2	

* *p* < 0.05, ** *p* < 0.001.

After adjusting for multiple newborn and maternal characteristics, season of birth and VitD related multivitamin supplementation remained associated with newborn VitD status. Newborns born in September through February were at a higher risk of VitD deficiency. The relative risk was 1.7 (95% CI, 1.1–2.6) in both autumn and winter (95% CI, 1.1–2.5) (*p* < 0.05). VitD or DHA supplementation more than once a day during pregnancy reduced the risk of VitD deficiency [adjusted OR (aOR) = 0.6, 95% CI (0.45–1.0) for VitD supplementation, and aOR= 0.7, 95% CI (0.51–0.95) for DHA supplementation] (*p* < 0.05) (Table 3).

**Table 3:** Factors associated with cord serum 25(OH)D < 20 ng/mL by multivariable analysis (*n* = 1030).

Variable	Crude OR	95% CI	*P*-value	aOR	95% CI	*P*-value
**Gestational age (weeks)**						
<37	1.0			1.0		
37–39	1.1	(0.55, 2.2)	0.778	1.0	(0.49, 2.3)	0.903
40+	1.4	(1.1, 1.9)	0.021*	1.3	(0.96, 1.8)	0.087
**Maternal age (years)**						
<30	1.0			1.0		
30–34	0.99	(0.75, 1.3)	0.917	0.98	(0.74, 1.3)	0.916
35–39	0.6	(0.36, 1.0)	0.052	0.61	(0.36, 1.1)	0.078
40+	0.42	(0.088, 2.0)	0.276	0.22	(0.026, 1.8)	0.156
**Maternal prepregnancy BMI**						
<28	1.0			1.0		
≥28	0.77	(0.43, 1.4)	0.395	0.75	(0.4, 1.4)	0.370
**Maternal education**						
Middle school or lower	1.0			1.0		
High school	0.64	(0.28, 1.4)	0.280	0.8	(0.32, 2.0)	0.615
College or higher	0.59	(0.28, 1.2)	0.161	0.73	(0.32, 1.7)	0.458
**Birth weight (Kg)**	1.0	(1.0, 1.0)	0.551	1.0	(0.98, 1.0)	0.738
**Month of birth**						
Summer (Jun.–Aug.)	1.0			1.0		
Autumn (Sep.–Nov.)	1.6	(1.1, 2.3)	0.017 *	1.7	(1.1, 2.6)	0.015*
Winter(Dec.–Feb.)	1.7	(1.2, 1.4)	0.015 *	1.7	(1.1, 2.5)	0.014*
**VitD supplementation during pregnancy**						
No	1.0			1.0		
≤6 times/week	0.57	(0.28, 1.1)	0.115	0.78	(0.35, 1.7)	0.552
≥1 time/day	0.52	(0.35, 0.75)	<0.001 **	0.6	(0.45, 1.0)	0.045
**Calcium supplementation during pregnancy**						
No	1.0			1.0		
≤6 times/week	0.86	(0.54, 1.4)	0.531	1.1	(0.63, 1.8)	0.817
≥1 time/day	0.72	(0.52, 1.0)	0.050 *	0.8	(0.56, 1.2)	0.243
**DHA supplementation during pregnancy**						
No	1.0			1.0		
≤6 times/week	0.61	(0.35, 1.1)	0.080	0.66	(0.36, 1.2)	0.197
≥1 time/day	0.67	(0.5, 0.9)	0.007 *	0.7	(0.51, 0.95)	0.022
**Outdoor activity in weekdays**						
<0.5 h	1.0			1.0		
≥0.5 h	0.93	(0.72, 1.2)	0.558	0.84	(0.58, 1.2)	0.371
**Outdoor activity in weekend**						
<0.5 h	1.0			1.0		
≥0.5 h	1.0	(0.81, 1.4)	0.720	1.3	(0.92, 1.9)	0.128
**Husband smoke during pregnancy**						
No	1.0			1.0		
Yes	1.2	(0.94, 1.6)	0.139	1.2	(0.9, 1.7)	0.184

# All parameter estimates were adjusted for other covariates. * *P* < 0.05.

## 4. Discussion

We found that 25(OH)D levels in cord blood from 1030 healthy Shanghai infants were quite low overall—almost 1/3 of the children had less than 20 ng/mL 25(OH)D in early life. Cord blood 25(OH)D level was positively correlated with summer birth and multivitamin supplementation containing VitD, two strong predictors of high VitD status.

A high prevalence of VitD insufficiency in newborns confirms that VitD insufficiency or deficiency is a major health problem in Shanghai. Our data, which showed that the median 25(OH)D level was 22.4 ng/mL with 36.3% of newborn participants having VitD deficiency and 84.1% having VitD insufficiency, was similar to that of Tao *et al*. [14] in a study of pregnant women in Shanghai (mean: 17.57 g/mL, deficiency: 69%, insufficiency: 91%). However, the prevalence of VitD insufficiency was much higher than that reported by a study of small sample size newborns in Beijing (insufficiency: 100%, *n* = 58) [12].

Serum 25(OH)D is the best indicator of VitD status [20]. Although a number of assays are now available for measuring 25(OH)D in the serum or plasma, recently liquid chromatography tandem mass spectrometry (LC–MS/MS) systems have been used for more rapid, specific and sensitive assessment [21] and are gaining wide-spread acceptance [22,23,24]. LC–MS/MS can accurately measure the different forms of VitD, including VitD_2_ and VitD_3_. In this study, the ratio of 25(OH)D_3_:25(OH)D_2_ was 4:1, which is similar to that reported by Karras *et al*. (3:1) [25]. In humans, VitD_3_ is produced from its precursor, 7-dehydrocholesterol, during exposure to ultraviolet rays contained in sunlight, or it can be consumed in the diet. The human body does not make VitD_2_, and the typically low level of VitD_2_ that is observed in humans is from dietary intake. Given that VitD_2_ is the only high-dose preparation of VitD available in many countries, potential differences in the ability of assays to accurately detect 25(OH)D_2_ and 25(OH)D_3_ are of clinical importance in cases where supplementation is suggested [25].

The seasonality of serum 25(OH)D levels in older children and adults has been well documented [26,27,28]. In contrast, few studies have examined this issue in newborns. Our study demonstrates seasonal variation in neonatal 25(OH)D levels. We found neonatal 25(OH)D levels were highest for newborns born from June to August and then decreased during the autumn and winter, from September through February. Our data are in accordance with two previous studies in New Zealand and Norway [29,30], but showed a relative weak seasonal variation. The lack of strong seasonal correlation could be due to the fact that Shanghai is an urban city with limited opportunities for exposure to the sun, and the Chinese women have the habit of avoiding exposure (umbrellas, hats, and sunscreen). Pregnant women also tend to stop working and stay indoors. VitD supplementation is also uncommon in China. In our study, only 21.3% of pregnant women took the VitD supplementation during their pregnancy, and they took a low dose at 200–400 IU/day.

Our study also showed that VitD related multivitamin supplementation during pregnancy was positively correlated with neonatal 25(OH)D levels. VitD fortified food is rare in China. Thus, VitD supplementation is the main source of VitD for pregnant women. DHA supplementation can also improve 25(OH)D level, possibly because VitD co-exists in DHA preparations. The Chinese Association of Obstetrics & Gynecology has not suggested any guideline for VitD supplementation for pregnant women. Accordingly, Chinese obstetricians do not pay any attention to the VitD status of pregnant women and few hospitals perform vitamin D detection. Thus, further research into VitD status and related health outcomes in China is urgently needed.

In addition, we did not find an association between VitD status and gestational age, especially in newborns born preterm. We speculate that this is because the prevalence of preterm deliveries was low (3.5%). This was a relative large cohort study of neonatal VitD levels. However, the present study has some limitations. We did not collect blood samples from the mothers. Thus, we could not measure maternal 25(OH)D levels, even though a strong positive association between maternal 25(OH)D and neonatal 25(OH)D has been well proven [31,32,33]. Notably, we are performing a follow-up study of the outcomes of the children in this study, including allergies and asthma.

## 5. Conclusions

In summary, VitD deficiency and insufficiency are common in newborns in Shanghai, China. At present, maternal VitD status is not a major concern in China. Our findings may help increase awareness of this problem and promote VitD supplementation during pregnancy to improve VitD status both in mothers and their newborn children.
